# VIM model: a novel model to depict the spatial heterogeneity of the radiation microenvironment

**DOI:** 10.7150/ijms.104046

**Published:** 2025-02-26

**Authors:** Yinan Sun, Ying Bi, Xiaohui Wang, Shunfang Liu, Lu Wang

**Affiliations:** 1 Department of Cardiology, Tongji Hospital, Tongji Medical College, Huazhong University of Science and Technology, Wuhan, Hubei Province, China.; 2 Department of Oncology, Tongji Hospital, Tongji Medical College, Huazhong University of Science and Technology, Wuhan, Hubei Province, China.; 3 The Center for Biomedical Research, Department of Respiratory and Critical Care Medicine, NHC Key Laboratory of Respiratory Diseases, Tongji Hospital, Tongji Medical College, Huazhong University of Science and Technology, Wuhan, Hubei Province, China.

**Keywords:** VIM model, radiation microenvironment, radiation-induced liver disease, spatial heterogeneity.

## Abstract

Radiation-induced disease (RID) is the most important factor limiting the radiotherapy dose for malignant tumors, especially for patients with organ insufficiency or chronic inflammation. In this paper, it studied the changes in the microenvironment after radiation exposure from the perspectives of molecular biology, cell biology and histopathology, and first proposed a novel model of the radiation microenvironment to depict radiation-induced spatial heterogeneity. The radiation microenvironment was divided into the VIM model: vascular microenvironment, inflammatory microenvironment, and metabolic microenvironment according to the special cell functions, molecular expressions and pathological structures after radiation. The structural functions of each microenvironment were explored to provide the new theoretical basis for molecular target prediction, radiation damage assessment, prevention and treatment of radiation-induced disease, and using radiation-induced liver injury as a template to depict the VIM model.

## 1. Introduction

Radiation-induced disease is caused by a dose of radiation exposure to the tissue with a series of pathological changes in local cells and nonparenchymal cells (NPCs)^1^. It is one of the most serious complications of radiotherapy. The occurrence of radiation-induced disease is related to the organ function status, the dose, volume, and segmentation method employed in radiotherapy^2^. For patients with tumors complicated by organ dysfunction, the prognosis of radiation-induced disease is poor and even life-threatening^3^. Although intensity modulated radiotherapy and stereotactic body radiotherapy have enabled patients to safely limit the organ volume, challenges remain for patients with advanced inflammation.

Taking the radiation-induced liver disease (RILD) as an example, among hepatic NPCs, Kupffer cells (KCs), hepatic stellate cells (HSCs) and hepatic sinus endothelial cells (HSECs) are sensitive to radiation^4^. Radiation damage to these cells *in vivo* can cause inflammation and apoptosis, and promote the production of inflammatory cytokines, forming a special inflammatory microenvironment (IME)^5^, ^6^. On the other hand, RILD leads to the loss of the normal structure and function of the biofilm, and eventually cell necrosis, disintegration and death, which will cause the loss of the biological function of the HCs, seriously interfere with the metabolic function of the liver and produce a special metabolic microenvironment (MME)^7^, ^8^. The hepatic blood sinuses around the central lobular vein of the irradiated liver tissue can be dilated and congested, and a large number of cytokines can activate HSCs to secrete extracellular matrix (ECM). It is deposited in the necrotic area, and the collagen fibers of the central lobular vein wall are deposited and thickened, or even completely occluded, eventually forming a typical hepatic veno-occlusive disease (VOD) and causing sequential changes in the vascular microenvironment (VME)^8^.

The development of the radiation microenvironment (RME) involves HCs, HSECs, KCs, HSCs, and various cytokines, such as tumor necrosis factor-α (TNF-α)^9^, transforming growth factor-β (TGF-β)^10^, and hedgehog (Hh)[11]signals. Based on this complex microenvironment, this paper is the first to propose the concept of a radiation microenvironment**.** According to spatial and biological functional heterogeneity, the RME is divided into the VME, IME amd MME called VIM model. The three types of microenvironments are not independent but participate in the construction of the RME together. A theoretical model of the RME and VIM model is introduced according to the characteristics of the pathophysiology, cell biology and molecular mechanism. It can expand the understanding of radiation and discover effective potential molecular targets, early biomarkers, and strategies to predict or treat radiation-induced disease, and improve radiotherapy efficacy.

## 2. Overview of RME and VIM model

**Figure 1:**
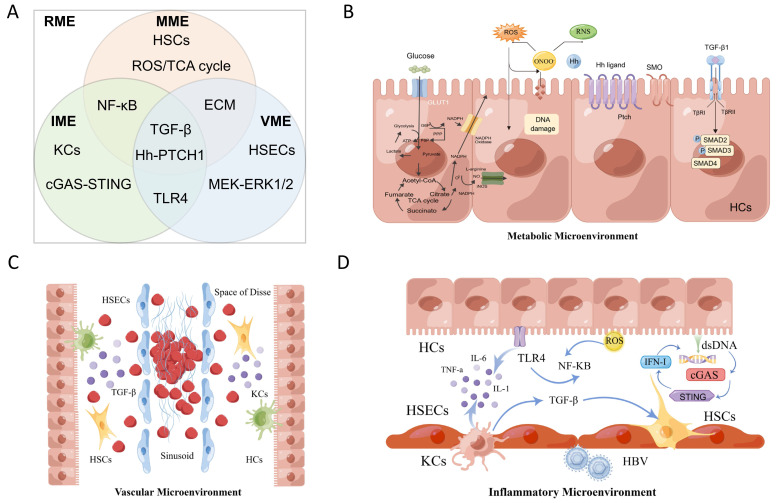
** According to spatial and biological functional heterogeneity, the RME is divided into the VME, MME and IME. (A)** The three types of microenvironments are not independent and participate in the construction of the RME together. **(B)** RILD leads to the loss of the normal structure and function of the biofilm and eventually cell necrosis, disintegration and death, seriously interfere with the metabolic function of the liver and produce the MME. **(C)** The hepatic blood sinuses around the central lobular vein of the irradiated liver tissue can be dilated and congested, even completely occluded, forming a typical hepatic veno-occlusive disease and causing sequential changes in the VME. **(D)** These induced cytokines activate KCs and other immune cells to form the IME.

The RME is the radiation microenvironment constructed by the continuous activation of TNF-α, TGF-β, Hh signals and complex multicellular responses associated with vascular alterations, collagen synthesis imbalance, and metabolic disorders after radiation^12^. It is essentially a radiation-related injury and repair response that constructs specific IME, VME, and MME,namely the VIM model, as shown in Figure 1. Molecular events of the RME involve complex and diverse biological processes, including aging, oxidative stress, inflammation, fibrosis and so on. The energy of radiation is transmitted or emitted in the form of waves or particles and can immediately produce highly active free radicals, leading to a radio adaptive response with DNA fragmentation, oxidative stress and an inflammatory response through molecular cytological processes activated by hepatic innate immune cells, recruitment of the circulating immune cell infiltration response, parenchymal cell loss and excessive formation of fibrous tissue^13^, resulting in distortion of hepatic structure and function during radiation, as is shown in Table 1.

## 3. The characteristics of the IME in RILD

Radiation-induced immune cells like KCs, blood circulation cells or bone marrow-derived immune cells, cytokines such as TGF-β, TNF-α, IL-1, and IL-6, and local immune responses such as the activation of reactive oxygen species (ROS), NF-κb, and cGAS-STING directly or indirectly construct the IME in the RME. Nuclear and mitochondrial DNA damage caused by direct exposure to radiation can lead to cell death through processes such as mitotic mutations, apoptosis, and primary or secondary necrosis^14^. Necrosis initiates the release of inflammatory cytokines such as TNF-α, IL-1β, and IL-6^15^, while apoptosis induces the release of anti-inflammatory cytokines such as TGF-β^16^. NPCs are induced to produce a large number of growth factors and chemokines, which promote the aggregation of inflammatory cells such as macrophages and lymphocytes, and RILD is eventually transformed into liver fibrosis due to the loss and repair process of HCs^17^.

### 3.1. The KCs are the key cells in the IME

KCs are macrophages that reside in the hepatic sinuses and are capable of recognizing, absorbing, and degrading cell debris or pathogens, and eliminating antibodies, debris, or dead cells, thereby ensuring the dynamic balance of the hepatic function^18^. KCs are also a major source of proinflammatory cytokines in radiation reactions that produce cytokines, including TNF-α, IL-1, and IL-6^19^. When the liver is damaged by external stimuli (such as radiation and bacteria), NPCs in tissues produce a large amount of ROS to kill bacteria and other substances and attract a large number of monocytes and macrophages into the liver by producing abundant inflammatory factors and chemokines^20^, ^21^. Infiltrated macrophages are thought to be more radiation-resistant than NPCs and not only promotes tissue repair, but also aggravates injury. Radiation-induced TNF-α release by KCs increases the susceptibility of HCs to radiation-induced apoptosis and ultimately leads to HCs death. The expression of TNF-α, IL-1 and IL-6 in radiation-induced macrophages is considered to mediate the pathogenesis of RILD^9^. After irradiation of KCs, TNF-α was released into the medium, and the apoptosis of HCs was significantly increased, suggesting that KCs secreted TNF-α, promoting HC apoptosis and leading to acute liver injury, and liver dysfunction^22^. In another study, KC-derived TNF-α and subsequent activation of TNFR1 in HCs promote RILD. GdCl3, a selective inhibitor of KCs, reduced radiation-induced production of IL-1, IL-6, and TNF-α and can improved acute RILD^23^.

### 3.2. The role of inflammatory pathways in the IME

The innate immune driving mechanism that amplifies the signals of cGAS-STING activation is involved in the formation of RILD^7^. Physical destruction of chromosomes or double-stranded DNA (dsDNA) of target cells by radiation is the main mechanism of cell death^24^. After apoptosis and necrosis of target cells, a large amount of genetic material dsDNA is released, which plays a role in normal cell damage^25^, ^26^. In particular, the liver, as a DNA-rich organ, can express up to 16 genome copies in mature HCs^27^. This damaging effect may become increasingly intense, resulting in a large amount of DNA deposition in the liver, triggering the DNA receptor cGAS-STING signals, and inducing more HCs death through paracrine and autocrine mediated cytotoxicity. Further release of dsDNA activates the cGAS-STING signal, forming a dsDNA-cGAS-STING-IFN-I positive feedback loop to induce a large amount of liver cell necrosis^28^. The increase in serum IFN-I concentration after radiation stimulation is related to the development of RILD in patients. Blocking IFN-I signals with drugs may prevent the occurrence of RILD. These results suggest that cGAS-STING-induced IFN-I release in NPCs of the liver is a key mediator of RILD and provide a strong basis for targeting cGAS-STING or inhibiting IFN-I signaling as a treatment or prevention method for RILD^29^.

Lobular blotchy necrosis and neutrophil infiltration around blood vessels in the liver is one of the manifestations of the early IME of RILD. Radiation can upregulate the expression of Toll-like receptor (TLR)4 in HCs and NPCs, and promote the activation of TLR4 signals^30^. Activation of TLR4 signals can promote the secretion of inflammatory factors, such as TNF-α, IL-1, and IL-6, and subsequently promote the infiltration of inflammatory cells, leading to liver inflammation and injury^31^. NF-κB plays a leading role in inflammation and can be activated by oxidative stress, interacting with inflammation^32^. In RILD, ROS activate NF-κB signals and proinflammatory cytokines^33^, anti-inflammatory agents and antioxidants may mitigate the development of RILD and reduce the expression of NF-κB. Radiation induces the activation of HSCs, and the activated HSCs undergo the TLR4 signaling cascade, promoting the secretion of inflammatory cytokines, chemokines and adhesion molecules into the cell culture medium. Inflammatory cells infiltrate into the damaged microenvironment, promoting the occurrence and development of the IME^34^.

## 4. The characteristics of the IME in RILD

ROS and active nitrogen induced by radiation can cause oxidative stress and peroxynitrite anions in tissues. This results in the imbalance of cellular endogenous enzymes and the reduction/redox system, as well as oxidative stress-induced metabolic reactions of intracellular proteins, lipids and nucleic acids. Consequently, it causes changes in cellular metabolic dysfunction and activates TGF-β and Hh signals constituting the radiation-related MME^35^.

### 4.1. The indicators of hepatic function in the MME

The liver is a major organ for carbohydrate and lipid metabolism, including digestion, absorption, transportation, catabolism and anabolism^36^. It is also involved in bile production, elimination of various metabolites, immunity, plasma protein synthesis and other physiological functions^37^. Radiation can directly lead to the destruction of bases and sugars in the DNA structure of HCs and NPCs. In addition, radiation can ionize water in liver tissue, forming oxygen radicals, hydroxyl groups, and peroxides. Free radicals further damage the liver tissue, resulting in the loss of the normal structure and function of the biofilm, eventually leading to cell necrosis and disintegration^38^. Bakshi et al. found that low-dose radiation could immediately inhibit the expression of pyruvate kinase, pyruvate dehydrogenase, dihydrolipamide S-acetyltransferase, ALDO-A and carnitine acetyltransferase, all of which are important enzymes in regulating glucose metabolism^39^. In addition, they found peroxisome proliferation-activated receptor-mediated metabolic alterations and late increases in cytochrome P450 enzyme levels^40^. After receiving low doses of radiation, mitochondrial ultrastructural damage and increased lipid deposition in HCs were observed, and another study found that after liver radiation, the content of several liver pentose circulating metabolites was increased, including glucose-6-phosphate, mannose-6-phosphate, and mannose-1-phosphate. Glucose-6-phosphate is involved in glycolysis, glycogen metabolism, and the oxidative branch of the pentose phosphate pathway (PPP)^41^.

### 4.2. TGF-β is the key marker in the MME

TGF-β plays an important role in regulating cell proliferation, differentiation, migration and fibrosis after injury^42^. It is also one of the most important fibrogenic cytokines in hepatic fibrosis^43^, and regulates the production, degradation and accumulation of ECM in RILD. Inflammatory cells, HSCs, and HSECs may be involved in the complex process of RILD as cellular sources of active TGF-β1^12^, ^44^, ^45^. TGF-β1 can induce fibrosis by activating both classical and nonclassical signals, leading to the activation of myofibroblasts, overproduction of ECM, and inhibition of ECM degradation^46^. Activated TGF-β1 phosphorylates and activates SMAD^46^ and directly bind gene promoters to induce transcription of fibroblast molecules, including cell tissue-smooth muscle actin, type I collagen and tissue matrix metalloproteinase inhibitor, leading to myoblast activation and matrix deposition. It is essential for the initiation and persistence of RILD. Hu et al. reported that paeoniflorin treatment can reduce RILD by inhibiting TGF-β-smad signals^47^. Xiao et al. also reported that HSCs activation can be triggered by TGF-β/PI3K/AKT signals after radiotherapy^48^. In the livers of irradiated rats, the expression level of TGF-β1 increased with increasing radiation dose, and this expression pattern was correlated with the degree of liver fibrosis. These fibrinogenic effects of TGF-β are mediated by excessive secretion of PAI-1, and plasminogen activator is one of the downstream targets of TGF-β, inhibiting plasminogen activator, thereby preventing the decomposition of fibrin and promoting the accumulation of fibrin, anti-TGF-β stimulation therapy is a strategy to treat the development of RILD.

### 4.3. The role of Hh signals in the MME

Hh signals are activated and involved in acute and advanced liver responses to radiation. Wang et al. showed that Hh signals were related to HSC activation, and Hh signal activity increased in response to RILD and induced compensatory proliferation of hepatic progenitor cells, myoblasts and HSCs, thereby promoting liver fibrosis^35^, ^49^. After 6 weeks of a single dose of radiation, the RNA expression of IHH (Hh ligand), SMO (Hh receptor) and GLI2 (Hh target gene) increased significantly. Triglyceride, TGF-β, α-SMA, and collagen I levels increased, and bone morphogenetic protein-7 levels decreased. At 10 weeks after single-dose radiotherapy, the expression of SHH (another Hh ligand), SMO and GLI2 RNA increased, and liver fibrosis occurred^35^. Similarly, Hh signals are upregulated in acute and chronic liver apoptosis and fibrosis in fractured radiation mouse models.

In addition, Hh inhibitors reduced Hh activity and attenuated hepatic progenitor cell or myofibroblast proliferation, liver damage, and fibrosis^50^. Therefore, Hh signals may play a potential role in the pathogenesis of RILD, liver fibrosis and liver regeneration after liver injury^35^. Hh signals have been identified as a potential factor involved in the liver's response to radiation. It was activated in early and late responses to segmented liver radiation in mice with early RILD and promoted progressive liver fibrosis. These results suggest that Hh signaling is a potential target for the treatment of RILD. Hh inhibitors are a potential strategy to modulate the liver radiation response.

## 5. The characteristics of the VME in RILD

**Figure 2:**
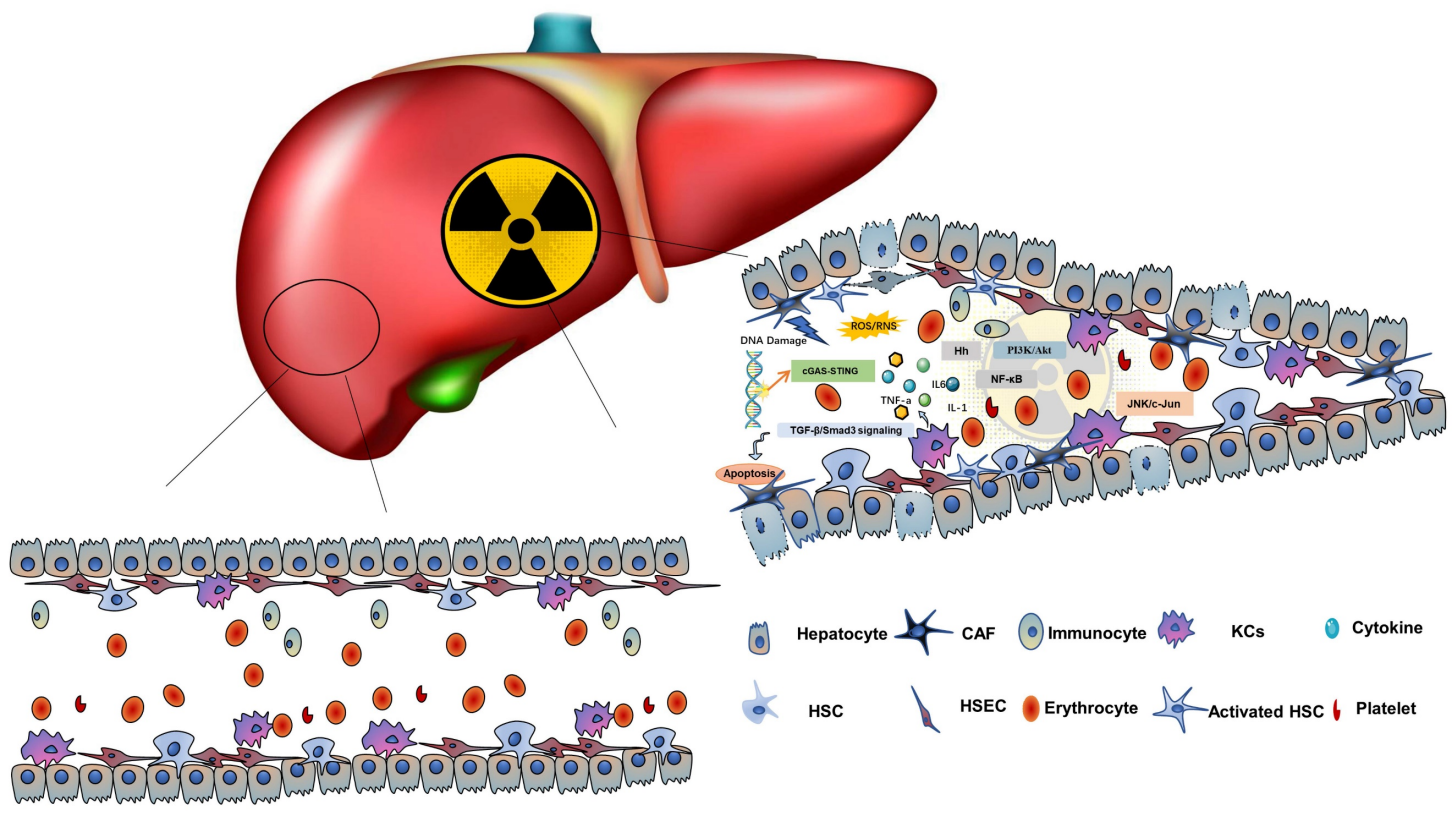
** The theoretical model of the RME.** Radiation damage to HCs and NPCs can cause inflammation, apoptosis and promote the production of inflammatory cytokines, forming the RME. It involves HCs, HSECs, KCs, HSCs, and various molecules, such as TNF-α, TGF-β, and hedgehog signals.

After liver radiation, blood microcirculation in hepatic sinuses and the dynamic regulation of ECM secreted by HSCs in the Disse space constitute the VME in the RME as shown in Figure 2. The typical pathophysiological manifestation of RILD is VOD, which is characterized by the entrapment of erythrocytes in the network of reticular proteins and collagen fibers, resulting in complete occlusion of the central venous cavity^51^-^53^. Trapped erythrocytes cause vascular congestion, resulting in reduced oxygen delivery to the central region. The hypoxic environment causes the death of lobular central HCs and atrophy of the intrahepatic plate, leading to liver dysfunction. When the central venous cavity is completely closed, the erythrocytes are surrounded by dense reticular and collagenous fibers that crisscross the lumen of the central vein, the inferior lobular vein, and the central hepatic sinus artery. There is a large loss of HCs in the center of the lobular cavity, possibly due to anoxic cell death secondary to vasocongestion. Approximately 4 months after injury, vascular congestion subsided, but there was persistent fibrosis in the portal, and central veins, and unrepaired lobular collapse in the injured area^54^.

### 5.1. The pathological dynamic changes in the VME

In the acute stage within 1 month after radiation, the hepatic sinusoid around the central lobular vein of the liver tissue can be dilated and hyperemic, and the HCs undergo balloon-like transformation and even necrosis. In the subacute stage, collagen fibers of the central lobular vein wall are deposited and thickened, or even completely occluded, usually within 1-3 months after radiation, thus forming VOD with collagenous fibers around the sink area, hepatic sinusoid, increased central venules and HC necrosis. In the chronic stage, six months to one year after radiation, a large amount of fibrous tissue hyperplasia in the liver, and HCs appear with flacked-like necrosis, hepatic sinusoid and further thickening of small blood vessels^55^. These are the pathological dynamic changes in the VME. Furthermore, excessive ECM induces HSECs to form basement membranes, cell proliferation and contraction increase intrahepatic sinus pressure, and multiple factors interact to lead to liver dysfunction, liver fibrosis and cirrhosis.

### 5.2. The endothelial cells are the key cells in the VME

Radiation can not only directly induce HCs but also HSECs apoptosis. HSECs injury can lead to microcirculation and blood flow disturbance, marginalization and activation of adhesion molecule-dependent white blood cells, and ultimately secondary HCs injury. Radiation-induced HSECs apoptosis and inflammation caused by oxidative stress are considered to be the initiators of RILD. Phenotypic changes in HSECs, capillaries, and basement membrane formation in the Disse space, have been shown to be precursors of fibrosis. Hepatic phlebitis is one of the pathological manifestations, characterized by the loss of affected venules ,sinus congestion, and the presence of an endothelium-mediated bystander effect. Cheng et al. found that apoptosis after radiation mainly occurred in HSECs, but not in HCs. *In vivo*, systemic infusion of MSC-conditioned medium can specifically inhibit HSECs apoptosis through the AKT and ERK1/2 pathways^56^, decrease the secretion of inflammatory factors and increase the expression of anti-inflammatory factors, all of which can improve the VOD of RILD. HCs and HSECs are separated by the perisinusoidal space, also known as the Disse space, which is a narrow gap between HCs and HSECs^57^. After HSCs activation, ECM and fibronectin EIIIA are secreted to form the basement membrane. It is an early change from RILD to radiation-induced liver fibrosis^58^.

## 6. Discussion

This study first proposed a theoretical model of a radiation-induced RME based on spatiotemporal pathological changes. According to the changes of hepatic function and structure caused by radiation, RME is divided into VME, IME and MME as the VIM model. Since there are few studies on microorganisms in RID, the microbial microenvironment is not discussed in detail. The VIM model summarizes the hepatic microenvironment induced by radiation, and propose molecular targets and drugs to predict, prevent and treat RID. Systematic theoretical research on RID has guiding significance for clinical radiotherapy practice.

RID is caused by radiotherapy for upper abdominal tumors, esophageal cancer, and pretreatment for bone marrow transplantation^59^-^61^. RID is a multistage, multistep dynamic process; it connects a series of reactions through a complex cascade network, where oxidative stress, inflammation, sensory, fibrotic, and immune responses interact under the regulation of multiple signaling pathways^62^, ^63^. Therefore, the theoretical model of the RME was divided into subtypes according to the differences in participating cells, signals and molecules, including the VME characterized by the structure of HSECs and blood sinuses, the IME was characterized by KCs, blood immune cells and inflammatory molecules, and the MME characterized by HSCs and the TGF-β, and Hh pathways^64^. Focusing on the damage, repair and reconstruction of HCs and liver tissues after radiation, the three groups responded to metabolism, inflammation and structure, and released various cytokines after radiation. The typical pathological manifestations of RILD are perivenous fibrosis, sinus obstruction, activation of KCs and HSCs, and injury to HCs^65^. The accumulation of toxicity caused by radiation leads to structural and functional abnormalities of the liver, even RILD and cirrhosis, which seriously affect subsequent treatment and prognosis.

Focusing on the RME and targeting the IME, VME and MME can expand the research direction of detection, prevention and treatment, and identify more potential biomarkers to further understand the molecular and cellular mechanisms in the future to promote the development of targeted radiotherapy and improve the quality of radiotherapy^66^. It lays a foundation for providing patients with economical, low-toxicity and efficient targeted drugs with radiotherapy.

## Figures and Tables

**Table 1:** The characteristics of the radiation microenvironment in radiation-induced liver injury.

	Inflammatory microenvironment	Metabolic microenvironment	Vascular microenvironment
Definition	The IME is a response triggered by radiation exposure, characterized by the involvement of diverse immune cells, cytokines, and local factors that collectively contribute to inflammation.	The MME is the altered cellular conditions within tissues caused by radiation-induced oxidative stress, leading to changes in cellular metabolic functions and activation of specific signaling pathways.	The VME refers to the complex changes occurring in the blood microcirculation within hepatic sinusoids, influenced by RILD.
Cells	KCs, LSECs, HSCs, NPCs	HSCs, ECs Fibroblasts, HCs	HSCs, SECs, HCs, LSECs, LSCs, LSICs, CVCs, Hematopoietic Stem Cells
Bio-factors	DNA damage, ROS, Growth factors and chemokines, Cytokines (TGF-β, TNF-α, IL-1, IL-6), HBV	ROS, RNS, Oxidative stress, DNA damage, Lipid peroxidation, liver dysfunction	VOD, Apoptosis, Oxidative stress, Cytokines (IL-6), ECM
Pathways	NF-κB, cGAS-STING, TLR4	TGF-β/SMAD, PI3K/Akt, Hh, Glucose metabolism, Lipid metabolism	Akt, ERK1/2, TGF-β, Hh

**IME**: inflammatory microenvironment; **MME**: metabolic microenvironment; **VME**: vascular microenvironment; **RILD**: Radiation-Induced Liver Disease; **KCs**: Kupffer cells; **HSCs**: Hepatic stellate cells; **LSECs**: Liver sinusoidal endothelial cells; **NPCs**: non-parenchymal cells; **ROS**: Reactive oxygen species; **HBV**: hepatitis B virus; **RNS**: reactive nitrogen species; **Hh**: Hedgehog; **ECs**: Epithelial Cells; **HCs**: Hepatocytes; **SECs**: Sinusoidal Endothelial Cells; **LSCs**: Liver Stem Cells; **LSICs**: Liver Sinusoidal Interstitial Cells; **CVCs**: central venous cells;** VOD**: venous occlusive disease; **ECM**: Extracellular Matrix;
